# Concentrations of Lead, Mercury, Arsenic, Cadmium, Manganese, and Aluminum in Blood of Romanian Children Suspected of Having Autism Spectrum Disorder

**DOI:** 10.3390/ijerph16132303

**Published:** 2019-06-28

**Authors:** Manouchehr Hessabi, Mohammad H. Rahbar, Iuliana Dobrescu, MacKinsey A. Bach, Liana Kobylinska, Jan Bressler, Megan L. Grove, Katherine A. Loveland, Ilinca Mihailescu, Maria Cristina Nedelcu, Mihaela Georgeta Moisescu, Bogdan Mircea Matei, Christien Oktaviani Matei, Florina Rad

**Affiliations:** 1 Biostatistics/Epidemiology/Research Design (BERD) core, Center for Clinical and Translational Sciences (CCTS), The University of Texas Health Science Center at Houston, Houston, TX 77030, USA; 2 Department of Epidemiology, Human Genetics, and Environmental Sciences, School of Public Health, The University of Texas Health Science Center at Houston, Houston, TX 77030, USA; 3 Division of Clinical and Translational Sciences, Department of Internal Medicine, McGovern Medical School, The University of Texas Health Science Center at Houston, Houston, TX 77030, USA; 4 Department of Child and Adolescent Psychiatry, University of Medicine and Pharmacy of Carol Davila, Psychiatry Clinical Hospital Alexandru Obregia, Bucharest, sector 4, 041914, Romania; 5 Human Genetics Center, School of Public Health, The University of Texas Health Science Center at Houston, Houston, TX 77030, USA; 6 Department of Psychiatry and Behavioral Sciences, McGovern Medical School, The University of Texas Health Science Center at Houston, Houston, TX 77054, USA; 7 Department of Biophysics and Cellular Biotechnology, University of Medicine and Pharmacy of Carol Davila, Bucharest, sector 5, 050747, Romania

**Keywords:** autism spectrum disorder, Romania, lead, mercury, arsenic, cadmium, manganese, aluminum

## Abstract

Environmental exposure to lead (Pb), mercury (Hg), arsenic (As), cadmium (Cd), manganese (Mn), and aluminum (Al) has been associated with neurodevelopmental disorders including autism spectrum disorder (ASD). We conducted a pilot study during May 2015–May 2107 to estimate blood concentrations of six metals (Pb, Hg, As, Cd, Mn, and Al) and identify their associated factors for children with ASD or suspected of having ASD in Romania. Sixty children, age 2–8 years, were administered versions of ADOS or ADI-R translated from English to Romanian. After assessment, 2–3 mL of blood was obtained and analyzed for the concentrations of the six metals. The mean age of children was 51.9 months and about 90% were male. More than half (65%) of the children were born in Bucharest. Over 90% of concentrations of As and Cd were below limits of detection. Geometric mean concentrations of Pb, Mn, Al, and Hg were 1.14 μg/dL, 10.84 μg/L, 14.44 μg/L, and 0.35 μg/L, respectively. Multivariable linear regression analysis revealed that children who were female, had less educated parents, exhibited pica, and ate cold breakfast (e.g., cereal), watermelon, and lamb had significantly higher concentrations of Pb compared to their respective referent categories (all *p* < 0.05 except for eating lamb, which was marginally significant, *p* = 0.053). Although this is the first study that provides data on concentrations of the six metals for Romanian children with ASD, the findings from this study could be useful for designing future epidemiologic studies for investigating the role of these six metals in ASD in Romanian children.

## 1. Introduction

Environmental exposure to several metals, particularly some heavy metals, has been associated with neurodevelopmental disorders [1,2,3,4]. For example, several studies have reported that perinatal or postnatal exposure to lead (Pb) is associated with poor neurodevelopmental outcomes in children, including lower intelligence quotient (IQ) and cognitive ability [5] and autism spectrum disorder (ASD) [6,7]. Children with neurodevelopmental disorders have also been found to have higher blood or urine concentrations of metals and metalloids, such as mercury (Hg) [1], arsenic (As) [8], cadmium (Cd) [9], manganese (Mn) [10,11], and aluminum (Al) [12].

ASD is a neurodevelopmental disorder [13,14] that may be associated with the exposure to metals [5,6,15,16], either alone or through gene-environment interactions [17] and epigenetic changes [18]. Some studies have reported that heavy metals, such as Pb, Hg, Al, As, and Cd are associated with ASD [6,15,16,19,20,21,22,23,24,25,26], while other studies found no such associations [27,28,29,30,31,32,33]. However, some of these studies did not have the necessary information to control for potential confounders [6,16]. For example, it is well-known that children with ASD have a higher incidence of gastrointestinal problems and, as a result, their diets are very different from those of typically developing (TD) children [34]. Furthermore, some studies did not explore potential interactions while assessing associations between environmental exposure to these metals and ASD. For example, Rahbar et al. [35] did not find an association between Mn and ASD based on additive models. However, they found a significant interaction between Mn and *glutathione S-transferase pi* 1 (*GSTP1)* in relation to ASD after controlling for a mixture of Pb, Hg, As, and Cd as well as several other covariates [36]. 

Limited data are available regarding ASD in Romania [37]. Although a 2012 report from the Romanian Ministry of Health indicated 7,179 diagnoses of ASD [38], experts estimate that there are at least 15,000 ASD diagnoses in Romania annually [38]. This discrepancy is because the frequency of ASD cases reported by the Ministry of Health do not account for ASD cases diagnosed by general practitioners and other physicians in the private sector [38]. Most regions of Romania have a poor service infrastructure, an education system ill-equipped to integrate ASD children, and limited availability of services for adults with ASD [38]. However, people in Romania may be at increased risk of neurodevelopmental disorders (e.g., ASD) and other health outcomes since they are exposed to a variety of environmental pollutants, including heavy metals [39,40,41,42,43,44,45]. Romania has many reported sources of pollution, including water, soil, and air pollution [39,40,44,45]. Mining activity in Romania has resulted in elevated levels of heavy metals in soil [46,47], which may have found their way into the food chain, particularly in locally sourced vegetables [48]. Romania has industrial areas that used heavy metals for extraction and processing of non-ferrous ores, resulting in long-term heavy metals pollution. Although the Romanian government has put in place some environmental protection measures (e.g., allowable limits) for heavy metals in soil, the soil in certain areas continues to contain 2–3 times the allowable limits for some of these metals, which can potentially contaminate fruits and vegetables. For example, leafy vegetables (e.g., lettuce) have been found to contain concentrations of Cd and Pb that are seven and 17 times greater than the maximum allowable limits, respectively, in Romania [49]. Another study, assessed levels of minerals and heavy metals in commercial fruit juices from Romanian markets (apple, peach, apricot, orange, kiwi, pear, pineapple, and multi-fruit) and reported that while concentrations of As and Cd in fruit juices were below the allowable limits, the Pb content in fruit juices (apple, peach, and orange) exceeded the allowable limits for drinking water [50] recommended by the World Health Organization (WHO) (2014) [51]. An assessment of soil contamination with heavy metals revealed that recommended safety threshold values are exceeded for As, Al, Pb, Mn, and Hg in 34 Romanian counties [40]. In addition, water contaminated with heavy metals is a major concern in the Tisa (Tisza) and Danube Rivers, and soil in areas near the Danube River Delta that is contaminated with heavy metals [44,45]. Pb was heavily exploited by mining industries in northwestern Romania, causing elevated lead levels in soil near mining and ore processing sites [47]. Moreover, in the central Romania Copsa Mica area, children are at risk of being exposed to Pb [46,52]. One study has reported adverse human health effects including saturnine encephalopathy (lead encephalopathy), radial nerve paralysis, and lead colic due to such exposures expressing concerns that Pb- or Cd-contaminated soils and vegetables may have significantly contributed to decreasing life expectancy of people living in the affected areas near Baia Mare and Copsa Mica in the last two decades [46].

To our knowledge, few papers have been published regarding concentrations of heavy metals in blood of Romanian children [53,54,55]. We planned a pilot case-control study to assess the feasibility of conducting a large-scale epidemiologic study in Romania for investigating the role of environmental exposure to six metals (Pb, Hg, As, Mn, Cd, and Al) and their interactions with select genes that are involved in contaminant metabolism in relation to ASD. Although the pilot project revealed difficulty in enrolling suitable TD children as controls, in this paper we use data from the 60 ASD cases to report concentration of the aforementioned six metals and identify factors associated with blood Pb levels in Romanian children with or suspected of having ASD.

## 2. Materials and Methods

### 2.1. General Description

In collaboration between faculty at University of Medicine and Pharmacy of Carol Davila (UMF Carol Davila) in Romania and the University of Texas Health Science Center at Houston (UTHealth), we conducted a pilot study to assess the feasibility of conducting long-term epidemiologic research focused on gene-environment interactions in relation to ASD in Romania. Currently, our study team in the department of Child and Adolescent Psychiatry at UMF Carol Davila diagnoses at least 1400 children per year who meet the Diagnostic and Statistical Manual of Mental Disorders, Fourth Edition (DSM-IV) [56] and International Classification of Diseases, 10th Revision (ICD-10) [57] criteria for ASD. From May 2015 to May 2017, after obtaining consents from the parents, sixty (60) children between ages 2–8 years who met the DSM-IV and ICD-10 criteria for ASD, were reevaluated based on translated versions of the Autism Disorder Observation Schedule (ADOS) [58] and Autism Diagnostic Interview—Revised (ADI-R) [59] in Romanian [60,61]. Those who exceeded the threshold of ADI-R domains (e.g., A) qualitative abnormalities in reciprocal social interaction; B) qualitative abnormalities in communication; C) restricted, repetitive, and stereotyped patterns of behavior; and D) abnormality of development evident at or before 36 months) [59] and ADOS domains (e.g., communication and social interaction domains (social affect score)) were classified as children with confirmed ASD. In our pilot study, 67% of children had confirmed diagnosis of ASD as they exceeded both ADOS and ADI-R thresholds, but the remaining 33% exceeded only one of these two thresholds (herein labeled as being “suspected of having ASD”). 

We also administered a socioeconomic status (SES) questionnaire to assess demographic characteristics, socioeconomic position, education level of the parents, and occupation of parents with a particular focus on potential exposure to metals, and family history of psychiatric disorders. A detailed food frequency questionnaire was administered to capture possible dietary exposure to metals and data were obtained about the frequency of the child’s weekly consumption of local and imported seafood, fruits and vegetables, meat/organ meat (e.g., liver), dairy products, eggs, grain/starches, peas, beans, nuts, juices/soft drinks, and sources of drinking and cooking water. In the food frequency questionnaire we asked the parents, how often their child ate certain foods and parents could select one of the following responses: I) Never or almost never, II) 1–3 times per month, III) Once per week, IV) 2–4 times per week, or V) 5 or more times per week. At the end of the interview, 2–3 mL of whole blood was collected from each child for assessment of exposure to the metals. The blood samples were stored at −20 °C at UMF Carol Davila, and then shipped to the Michigan Department of Health and Human Services (MDHHS) in Lansing, MI, USA, for analysis of the six metals. Institutional Review Boards (IRBs) of UTHealth (Project identification code: HSC-GEN-15-0844) and UMF Carol Davila (Project identification code: PO-35-F-03) approved this study.

### 2.2. Methods for Assessment of the Metals Exposure

For this study, assays for trace metals (Pb, Hg, As, Cd, Mn, and Al) were performed at MDHHS Trace Metals Laboratory, a facility certified by the Centers for Disease Control and Prevention (CDC). Venous whole blood samples were diluted and analyzed for trace metals using a PerkinElmer Elan DRCII inductively-coupled plasma mass spectrometer (PerkinElmer, Waltham, MA, USA). The methods for analysis of the six metal concentrations in blood by MDHHS have been described previously [62,63]. 

The limits of detection (LoD) for Pb, Mn, Al, Hg, As, and Cd were 0.25 μg/dL, 2.5 μg/L, 5.0 μg/L, 0.25 μg/L, 1.3 μg/L, and 0.13 μg/L, respectively. All Pb and Mn concentrations were above the LoD. The percentage of concentrations below LoD for Al, Hg, As, and Cd were 30%, 61.7%, 90%, and 95%, respectively.

### 2.3. Statistical Analysis

We conducted descriptive analyses to examine the distributions of various characteristics of the study population including demographics and SES. Since the distributions of blood concentrations of Pb, Hg, Mn, and Al were skewed, we transformed these data using the natural logarithm (ln) in order to produce distributions that better approximated a normal distribution. The means of the ln-transformed blood concentrations of the four metals were transformed to their original scale (i.e., μg/dL for Pb and μg/L for the other metals) by applying an exponential function, herein called geometric mean. We also report the arithmetic (i.e., untransformed) means for comparative purposes. For Al and Hg concentrations below the LoD were replaced by the LoD for that metal divided by the square root of two (i.e., 2^−1/2^LoD) [64]. Since over 90% of concentrations of As and Cd were below LoD, we did not report arithmetic or geometric mean concentrations for these metals.

For Pb, Mn, and Al, which had more than 70% concentrations above LoD, we used univariable and multivariable General Linear Models (GLMs) to identify factors associated with concentrations of these metals. For GLM analysis, we categorized all responses in the food frequency questionnaire into binary variables “never consumed” = “no” or “consumed” = “yes”. Potential factors considered included gender, education of parents, distance of residence from a major road, and binary consumption of various kinds of seafood or fish, leafy vegetables (e.g., lettuce), fruits (e.g., watermelon), meat (e.g., lamb), grains (e.g., cereal for breakfast), and nuts. All variables that were significantly (i.e., *p* < 0.05) or marginally (i.e., 0.05 ≤ *p* <0.10) associated with metals in univariable analysis were entered in multivariable GLMs. As a result, we could not report significantly associated factors from multivariable GLMs for Mn and Al. In addition, for Hg, As, and Cd, which had more than 65% of concentrations below LoD, we did not identify factors associated with these metal concentrations. All statistical tests were performed at 5% level of significance. All statistical analyses were conducted using SAS 9.4. [65]. 

## 3. Results

### 3.1. Characteristics of Study Sample

The mean age of children was 51.9 months and about 90% were male. More than half (65%) of the children were born in Bucharest, and 45.5% of fathers and 71.7% of mothers had education beyond high school. Nearly 48% of children had family history of different psychiatric or neurodevelopment disorders as shown in Table 1. Demographic and SES characteristics are displayed in Table 1.

### 3.2. Arithmetic and Geometric Means and Median Blood Metal Concentration of ASD Children

Geometric mean (GM) concentrations of Pb, Mn, Al, and Hg were 1.14 μg/dL, 10.84 μg/L, 14.44 μg/L, and 0.35 μg/L, respectively. Arithmetic mean concentrations of these metals are also reported in Table 2. Percentage of children with elevated levels of the metals was 5% for Pb; 17% for Mn; 60% for Al, 3.3% for Hg; 0% for As; and 1.7% for Cd, based on cut-off values recommended by the Agency for Toxic Substances and Disease Registry (ATSDR) [66,67,68,69,70], Centers for Disease Control and Prevention (CDC) [71], and Laboratory Corporation of America (LabCorp) [72], reported in Table 2. 

### 3.3. Factors Associated with Blood Lead Concentrations Based on Univariable and Multivariable General Linear Models

Univariable linear regression analysis revealed that, compared to boys, girls had a significantly higher concentration of Pb (GM of 2.48 μg/dL vs. 1.05 μg/dL, *p* < 0.01). Children with less educated parents had a significantly higher mean concentration of Pb than those whose parents had higher education (GM of 1.78 μg/dL vs. 0.96 μg/dL, *p* < 0.01). Those who exhibited pica (i.e., eating mud) had a significantly higher mean concentration of Pb than those who did not (GM of 3.44 μg/dL vs. 1.01 μg/dL, *p* < 0.01). Residential proximity to high traffic roads was also associated with blood Pb concentrations; children living near the roads (i.e., within a quarter of a mile) had significantly higher concentration of Pb compared to children living further away from the roads (GM of 1.47 μg/dL vs. 0.98 μg/dL, *p* = 0.04). Compared to children who did not eat the following foods, respectively, children who ate these foods had a significantly higher blood concentrations of Pb; lettuce (GM of 1.50 μg/dL vs. 0.99 μg/dL, *p* = 0.04); watermelon (GM of 1.40 μg/dL vs. 0.87 μg/dL, *p* = 0.02); lamb (GM of 1.84 μg/dL vs. 1.05 μg/dL, *p* = 0.04); cold breakfast such as cereal (GM of 1.32 μg/dL vs. 0.84 μg/dL, *p* = 0.03); peanuts (GM of 1.49 μg/dL vs. 0.89 μg/dL, *p* < 0.01); tree nuts (GM of 1.53 μg/dL vs. 0.92 μg/dL, *p* < 0.01); and oil from animal fat (e.g., used for cooking) (GM of 2.02 μg/dL vs. 1.07 μg/dL, *p* < 0.05). Other univariable results are reported in Table 3.

In the final multivariable model that included all variables significantly associated with Pb in univariable analysis as described earlier, the following variables were identified as independently associated with having higher levels of Pb in the presence of other variables in the model: child’s sex (GM of 2.58 μg/dL for girls vs. 1.52 μg/dL for boys, *p* = 0.03), children of parents with lower education (GM of 2.48 μg/dL vs. 1.58 μg/dL, *p* < 0.01), children who exhibited pica (GM of 2.68 μg/dL vs. 1.46 μg/dL, *p* = 0.01), children who ate cold breakfast such as cereal (GM of 2.67 μg/dL vs. 1.46 μg/dL, *p* < 0.01); and children who ate watermelon (GM of 2.38 μg/dL vs. 1.64 μg/dL, *p* < 0.01). Consumption of lamb became marginally significant (*p* = 0.05) in the final multivariable model as shown in Table 3.

### 3.4. Factors Associated with Blood Manganese Concentrations Based on Univariable General Linear Models

Univariable linear regression analysis for Mn revealed that children who ate the following foods had a significantly higher geometric mean concentration of Mn compared to those who did not eat these foods: fresh shellfish (lobster, crab, crawfish) (GM of 20.00 μg/L vs. 10.72 μg/L, *p* = 0.05); banana (GM of 11.13 μg/L vs. 9.10 μg/L, *p* = 0.09); and polenta (GM of 11.48 μg/L vs. 9.88 μg/L, *p* = 0.07), though two of these associations were marginally significant as shown in Table 4. When all of these variables were entered in a multivariable linear regression model, none of these factors were significantly associated with Mn concentrations at 5% level of significance.

### 3.5. Factors Associated with Blood Aluminum Concentrations Based on Univariable General Linear Models

Similarly, in univariable analysis for Al, children who ate the following foods had significantly higher Al concentrations compared to those who did not eat these foods: cantaloupe or honeydew (GM of 20.47 μg/L vs. 10.86 μg/L, *p* = 0.04); shrimp (GM of 44.95 μg/L vs. 13.02 μg/L; *p* = 0.03); and lamb (GM of 32.06 μg/L vs. 12.54 μg/L, *p* = 0.03) as shown in Table 5. Again, when these variables were entered in a multivariable linear regression model, none of these factors were significantly associated with Al.

## 4. Discussion

A limited number of studies have been published regarding concentrations of the heavy metals in blood and teeth of Romanian children [53,54,55]. Nicolescu et al. investigated associations between environmental exposure of Romanian children to neurotoxic metals (Pb, Hg, and Al) and attention-deficit hyperactivity disorder (ADHD) [73]. To our knowledge, our study is the first study that reports concentrations of the six heavy metals in blood of children with ASD or suspected of having ASD in Romania. It is important to note throughout the discussion section that our results may not be generalizable to the general population of Romanian children due to the presence of a neurodevelopmental disorder. Additionally, we can reasonably expect that children with ASD may have different dietary exposures than children in the general population due to the high rate (23%–70%) of comorbid gastrointestinal disorders among children with ASD [74,75]. As a majority of the relevant literature is focused on exposures to Pb, in the following sections we will discuss Pb separately from the other five metals.

### 4.1. Blood Lead Concentrations and Their Associated Factors

In the 1980s, the Environment and Health Information System (ENHIS) study, a collaborative study between the World Health Organization and the European Commission, reported that geometric mean concentrations of Pb in children’s blood ranged from 18.2–18.9 μg/dL in Bulgaria, Hungary, and Romania, which were much higher than the concentrations of blood Pb in children from other countries in Europe such as Italy (11.0 μg/dL) and Germany (7.4 μg/dL) [53]. In 1998, the World Bank reported that many Central and Eastern European countries had gradually reduced the maximum allowed concentration of lead in gasoline to the current European Union standard (0.15 g/L), however Romania still allowed lead concentrations of up to 0.6 g/L [76]. After phasing out the use of lead in gasoline, the Pb blood concentrations in European countries dropped. For example, Romania phased out the use of lead in gasoline in 2005, which may partially explain why Nicolescu et al. reported lower arithmetic blood Pb concentrations of children with ADHD in 2006 in Bucharest (3.2 μg/dL) and Pantelimon (5.1 μg/dL), (a city near a metal-processing plant) [73] compared with the levels reported by WHO for Romania in the mid-1980s [53]. In the present study, our results from the univariable models revealed that children who have been living within quarter of a mile of a high traffic road had significantly (*p* = 0.04) higher mean blood Pb concentrations than those lived further away. On the other hand, we found relatively lower geometric mean concentrations of Pb in blood of children with ASD in Romania (1.14 μg/dL) compared to those reported by Nicolescu et al. [73]. Furthermore, the percentage of children with elevated levels of Pb (i.e., >5 μg/dL) in our study was 5%, lower than 25% reported by Nicolescu et al. [73]. The difference between the mean blood Pb concentrations and percentages of elevated levels of Pb in our study and that of Nicolescu et al., could be due to the areas where the sampled children live. Specifically, Nicolescu et al. stated that their study samples were collected from children in Bucharest (44.6%) and from a more Pb contaminated industrial area of Pantelimon, about 2 km south-east of Bucharest (55.4%), whereas in our study a majority (65%) of children were from Bucharest. Overall, our study and Nicolescu et al. [73] provide important information indicating that lowering lead in gasoline may have had a significant effect in reducing blood Pb concentrations among children in Romania. However, additional interventions may be needed to eliminate sources of Pb exposure through air pollution.

Significantly higher concentrations of Pb in children from the present study who ate lettuce (GM of 1.50 μg/dL vs. 0.99 μg/dL, *p* = 0.04) is consistent with a report that showed high levels of Pb in leafy vegetables (lettuce, parsley, dill, and orach) produced in Romania [49]. Industrial areas located near the south-east region of Bucharest, which has higher Pb concentrations in soil, could affect blood Pb concentrations of children living nearby [77]. This point was also emphasized by a study from Romania (Deliea et al.) that assessed Pb concentrations in children’s teeth and reported that the levels were 48% higher for children living in industrial areas, (Slatina city with high level of pollution) when compared to the reference region (Pătârlagele, a small village in a mountainous region with lower level of pollution) [55]. A more recent study from Romania in 2015 by Brad et al. also reported higher median concentration of Pb in blood (3.3 μg/dL (ranging from 1.3–13.0 μg/dL)) in contaminated areas that have mining activities, in north Romania [54]. As a result, the Romanian government could raise awareness of people that are living in these areas. 

In this study, we found that girls had a significantly higher concentrations of Pb compared to boys (GM of 2.48 μg/dL vs. 1.05 μg/dL). While there are currently no comparable studies from Romania, there are studies from neighboring countries including Poland and Italy that investigated the association of sex and blood Pb concentrations and reported findings different from our own. For example, a study, conducted in 2009 in Poland, reported no significant differences in blood Pb concentrations between boys and girls [78], while another study, conducted in 2012 in Poland, reported a slightly higher geometric mean concentration of blood Pb for boys than girls (GM of 2.54 μg/dL vs. 2.39 μg/dL), though this difference was only marginally significant (*p* = 0.08) [79]. A study from Italy carried out during 1993–1994 also reported no significant difference between blood lead concentrations in boys and girls [80]. One possible reason for the differences between our results and the Polish and Italian studies is that, our study included children with ASD, which resulted in having a much higher ratio of boys to girls (9:1) than would be expected in the general population. We also had a very limited sample size (only six girls), which warrants further replication of our finding. 

Parental education was another factor associated with blood lead concentrations in the present study; we found that children who had less educated parents, had significantly higher concentrations of Pb compared to children whose parents had higher education (GM of 1.78 μg/dL vs. 0.96 μg/dL). In the previously mentioned Polish study, Barrett reported no significant difference in mean blood Pb concentrations between children based on the education levels of their parents [78]. 

Our finding that children who exhibited pica had a significantly higher blood concentration of Pb than those who did not exhibit pica is consistent with two other studies in India and Pakistan. [81,82]. These findings are potentially important for Romanian children since, as discussed earlier, lead levels in soil are elevated above the maximum allowable limits in many areas across Romania.

In our univariable analysis, we found that children who ate lamb had a higher blood Pb concentration, compare to those who did not eat lamb. However, in the multivariable model this association became marginally significant. Based on other studies, lead poisoning affects livestock of all ages. Another possible source of exposure to Pb in farm livestock could be from their habit of chewing disposed batteries or eating items contaminated with used oil from machinery [83]. In addition, we found that children who ate watermelon had a significantly higher blood lead concentrations. Since Romania is ranked twenty-sixth in terms of production of watermelon in the world (https://www.tridge.com/intelligences/watermelon/RO/production) and contributes 0.4% share of world imports, our finding has revealed a specific source of exposure to lead in Romanian children. Moreover, in the multivariable model we found children who ate cold breakfast such as cereal had a significantly higher geometric mean concentration of lead in blood (GM of 2.67 μg/dL vs. 1.46 μg/dL, *p* < 0.01), compared to those who did not eat such breakfast. Although we did not find any other studies that reported a significant association between eating cereals and higher Pb concentrations in blood of children, a recent study from Australia, reported that levels of Pb found in breakfast cereals, rice products and diets of a selected group of children in Brisbane, ranged from <0.01 to 0.25 mg/kg. The estimated daily intake of Pb in children varied widely and ranged from 0.90 to 11.7 (5.6 ± 3.5 μg/day) [84]. Since lead is found in soil of some parts of Romania [46,47], we suggest further investigation of the Pb in cereal marketed in Romania. 

### 4.2. Blood Concentrations of Other Metals

The literature on blood concentrations of Mn, Al, Hg, Cd, and As in children from Romania and surrounding countries is very limited. In the present study, we found that the geometric mean blood concentrations of Mn in Romanian children with ASD is 10.84 μg/L. This is much higher than the concentrations reported in a study on adults from Italy (1.35 µg/L) [85]. 

We reported that geometric mean concentrations of Al in blood of Romanian children with ASD is 14.44 μg/L, which is lower than the previously mentioned study on Romanian children with ADHD (36.0 μg/L to 49.0 μg/L) [73] but much higher than a study on TD Italian children (1.49 µg/L in serum) [85]. The reasons for such a large difference may need to be investigated in future studies. On the other hand, based on recommended cut-off value of 9.0 μg/L for elevated blood Al concentration reported in Table 2, our result shows that the percentage of children with elevated levels of Al was 60%, much lower than that of a study on Romanian children with ADHD that was nearly 99% [73].

In this study, we found that the geometric mean concentration of Hg in blood of Romanian children with ASD was 0.35 μg/L and 3.3% of children had elevated Hg concentrations (i.e., >5 μg/L). Nicolescu et al. reported lower arithmetic blood Hg concentrations of children with ADHD in Romania (0.1 μg/L), and none of the children had elevated concentrations [73]. National European Surveys and other studies reported geometric mean blood mercury concentrations for many countries in Europe [86]. Our results from Romania are comparable to results from TD children (8–10 years old) in the Czech Republic in 2008 and German TD children (3–14 years old) during 2003–2006, who have geometric mean blood Hg concentrations of 0.45 µg/L and 0.23 µg/L, respectively. In contrast, our results were about half of that reported in Italian TD children (13–15 years old) during 2008–2010 (0.84 µg/L) [87,88]. 

In our study, since 95% of concentrations for Cd were below LoD, we did not report the geometric and arithmetic means. However, percentage of elevated levels for Cd was 1.7%. Our finding suggests lower concentrations of Cd than levels reported in a 2012 Polish study on preschool children living near environmental hazards (0.25 μg/L for boys and 0.24 μg/L for girls) [79]. Additionally, a 1998 study on people living in mining areas of Seville, Spain, reported a geometric mean of blood Cd concentration of 0.19 µg/L in people living close to the spill of toxic material from mining, which was higher than that of the unaffected population (0.14 µg/L) [89]. 

Similarly, we did not report the geometric and arithmetic means for As due to 90% of concentrations being lower than LoD. As a result, none of children in our study had elevated levels for As. On the other hand, we did not find blood As concentrations for children in other European countries to compare our results with. However, in our Jamaican autism study, we found that geometric mean blood arsenic concentration for Jamaican children with ASD was 2.48 μg/L, much higher than that of Romanian children with ASD. 

## 5. Limitations

Despite interesting findings reported from this pilot study, we acknowledge that this study has several limitations. First, this study was a pilot study with limited funding. Although our Romanian partners could have enrolled a larger number of children with ASD, shipment of the specimens to the US and assessment of the six metals would have not been possible for more than 60 children. Second, the sample size of 60 children in this study may not provide sufficient statistical power for investigating factors associated with concentrations of metals, especially those that had a sizable proportion below LoD. Third, blood may not be the most suitable biomarker for assessing exposures to all six of these metals. For example, urine is considered as a better biomarker for assessment of exposure to As [67] and Cd [90]. Hair is usually used for assessment of Hg [91] and Mn [92]. However, blood is considered as a good biomarker for assessment of Pb [93] and Al [94]. Fourth, due to 50% missing data regarding parents’ specific occupation, we did not report information regarding occupation of parents as a potential source of exposure to the metals. Fifth, the sample of 60 children with ASD or suspected of having ASD may not be representative of children from Romania due to the presence of a neurodevelopmental disorder. Additionally, the children in our study were enrolled primarily in and around Bucharest. It is also important to keep in mind that the type I error of 5% is for testing individual factors associated with Pb and does not account for multiple testing performed in this study.

## 6. Conclusions

This is the first study from Romania reporting concentrations of the six heavy metals in blood of children with ASD or suspected of having ASD. Considering that Romania has higher levels of some of these metals in soil, this study provides important information regarding concentrations of these metals in blood of potentially exposed Romanian children. We have also identified factors associated with concentrations of Pb in blood of children with ASD or suspected of having ASD, including dietary factors. These factors include child’s sex, parental education, exhibiting pica, and eating watermelon, lamb, and cold breakfast such as cereal. The findings from this study may help with designing relevant interventions to reduce concentrations of Pb in blood of Romanian children. These findings also provide important information for designing future epidemiologic studies, particularly those focused on assessing the role of these six metals in ASD among children in Romania.

## Figures and Tables

**Table 1:** Characteristics of children with ASD (or suspected of ASD) and their parents (*N* = 60).

Variables	Categories	*n* (%)
Child’s sex	Male	54 (90.0)
Child’s age (months)	Age < 48	32 (53.3)
	48 ≤ Age < 72	18 (30.0)
	Age ≥ 72	10 (16.7)
Child’s race	Caucasian (white)	59 (98.3)
	Other	1 (1.7)
Child’s ethnicity	Romanian	59 (98.3)
	Roma	1 (1.7)
Maternal age (at child’s birth) ^a^	< 35 years	50 (87.7)
	≥ 35 years	7 (12.3)
Paternal age (at child’s birth) ^b^	< 35 years	42 (71.2)
	≥ 35 years	17 (28.8)
Maternal race ^c^	Caucasian (white)	59 (98.3)
Paternal race	Caucasian (white)	59 (98.3)
	Other	1 (1.7)
Maternal education (at child’s birth) ^d^	Up to high school *	15 (28.3)
	Beyond high school **	38 (71.7)
Paternal education (at child’s birth) ^e^	Up to high school *	30 (54.5)
	Beyond high school **	25 (45.5)
Socioeconomic status (SES)	Car ownership	11 (18.3)
	Home ownership	41 (68.3)
Family history of neurodevelopment disorders ^f^	Autism spectrum disorder (ASD)	4 (6.7)
	Attention-deficit/hyperactivity disorder (ADHD)	7 (11.7)
	Learning problems	8 (13.3)
	Speech problems	19 (31.7)
	Behavior or disruptive problems at school	5 (8.3)
	Difficulties interacting with other people	3 (5.0)
	Intellectual disability	2 (3.3)
	Seizure	5 (8.3)

* Up to high school education means attended Primary/Jr. Secondary, and Secondary/High/Technical schools; ** Beyond high school education means attended a Vocational, Tertiary College, or University. ^a^ Maternal age was missing for three mothers. ^b^ Paternal age was missing for one father. ^c^ Maternal race was missing for one mother. ^d^ Maternal education was missing for seven mothers. ^e^ Paternal education was missing for five fathers. ^f^ Family history includes; mother, father, siblings, grandparents, uncles, aunts, cousins, nieces or nephews.

**Table 2:** Arithmetic and geometric mean blood metal concentration of ASD children (*N* = 60).

Variables	LoD	% Below LoD	Arithmetic Mean	Median	Geometric Mean ^a^	Recommended Cut-Off Level	Percentage above the Cut-Off Levels
Lead (μg/dL)	0.25	0	1.55	1.10	1.14	5	5
Manganese (μg/L)	2.5	0	11.41	10.50	10.84	15	17
Aluminum (μg/L)	5.0	30	29.77	14.00	14.44	9	60
Mercury (μg/L)	0.25	61.7	0.82	0.25	0.35	5	3.3
Arsenic (μg/L)	1.3	90	NR	NR	NR	12	0
Cadmium (μg/L)	0.13	95	NR	NR	NR	0.315	1.7

^a^ Mean blood metal concentration indicates the geometric mean = Exp. [Mean (ln metal concentration)]. NR: Geometric and arithmetic means as well as medians are not reported, because over 90% of As and Cd concentrations in blood were below LoD.

**Table 3:** Factors associated with blood lead concentrations based on univariable and multivariable General Linear Models (*N* = 60).

Exposure Variables	Category		Univariable					Multivariable ***		
			Yes		No		*p*-Value	Yes	No	*p*-Value
			Geometric Mean Pb (μg/dL) *	*N*	Geometric Mean Pb (μg/dL) *	*N*		Adjusted Geometric Mean Pb (μg/dL) **	Adjusted Geometric Mean Pb (μg/dL) **	
Sex of child	Male		1.05	54	2.48	6	0.006	1.52	2.58	0.03
Maternal age *^a^* (at child’s birth)	More than 35 years		0.97	7	1.17	50	0.53			
Paternal age *^b^* (at child’s birth)	More than 35 years		1.45	17	0.99	42	0.06	-	-	-
Parental education levels *^c^* (at child’s birth)	At least one of the parents had education beyond high school		0.96	36	1.78	14	0.005	1.58	2.48	0.004
Socioeconomic status (SES)	High SES (own a car)		1.17	11	1.14	49	0.92			
Source of drinking water *^d^*	Bottled water		1.07	51	0.93	3	0.74			
Source of cooking water *^e^*	Piped water		0.56	47	1.15	5	0.03			
Pica (habitually put items in mouth)	Mud		3.44	6	1.01	54	<0.0001	2.68	1.46	0.01
Living near a high traffic road *^f^*			1.47	24	0.98	35	0.04	-	-	-
Home environment	Living with adults whose jobs involve construction		1.16	10	1.14	50	0.94			
Types of toys the child plays with	Plastic		1.13	59	2.80	1	0.23			
	Electronic		1.03	44	1.53	16	0.07			
	Battery operated		1.07	51	1.66	9	0.11			
	Stuffed		1.20	15	1.12	45	0.78			
Types of pots, pans, and dishes used at home	Cast iron *^g^*		1.46	18	1.02	41	0.10	-	-	-
	Teflon		1.16	55	0.96	5	0.59			
	Aluminum *^h^*		1.22	29	1.06	29	0.49			
Fruits and vegetables consumption	Leafy vegetables	Lettuce	1.50	21	0.99	39	0.04	-	-	-
		Spinach, kale	0.94	29	1.34	31	0.07			
	Fruits	Blackberry	0.77	13	1.27	47	0.03			
		Banana	1.11	52	1.41	8	0.40			
		Watermelon	1.40	34	0.87	26	0.02	2.38	1.64	0.008
		Other melons (cantaloupe, honeydew)	1.22	27	1.08	33	0.54			
Meat consumption	Beef		0.99	42	1.59	18	0.02			
	Lamb		1.84	9	1.05	51	0.04	2.37	1.65	0.053
	Goat		2.23	4	1.09	56	0.6	-	-	-
	Pork		1.22	44	0.95	16	0.26			
	Minced meat (beef/pork/chicken)		1.27	47	0.78	13	0.04	-	-	-
Grain/Starches consumption	Dark bread		0.88	21	1.31	39	0.05			
	Oatmeal		0.94	10	1.19	50	0.38			
	Cold breakfast (cereal)		1.32	41	0.84	19	0.03	2.67	1.46	0.0001
	Pasta, macaroni, noodles		1.06	50	1.67	10	0.08			
	Biscuits		1.17	59	0.28	1	0.06	-	-	-
	Polenta		1.22	37	1.04	23	0.42			
Peas, beans, and nuts	Red beans		1.04	8	1.16	52	0.71			
	Peanuts, cashews		1.49	29	0.89	31	0.008	-	-	-
	Tree nuts (pecans, walnuts)		1.53	26	0.92	34	0.007	-	-	-
Do you use oil from animal fat for cooking?			2.02	6	1.07	54	<0.05	-	-	-

* Mean Pb indicate the geometric mean = Exp. [Mean (lnPb)]; ** Adjusted for sex, parental education levels, pica (habitually put items in mouth), ate watermelon, consumed lamb, and ate cold breakfast (cereal); *** Levels of parental education was missing for 10 parents. As a result the sample size for multivariable analysis was based on *N* = 50. The **Yes** column includes children who met the category specified in front of each exposure variable or the food consumed. The **No** column includes children who did not meet the category specified in front of each exposure variable or never consumed the food. “-“ Indicates exposure variables that had *p*-value < 0.10 in univariable analysis but were not significantly associated blood metal concentrations in multivariable models at 5% level. **^a^** Maternal age was missing for three mothers. ^b^ Paternal age was missing for one father. *^c^* Parental education levels were missing for 10 parents. *^d^* Source of drinking water was missing for six households. *^e^* Source of cooking water was missing for eight households. *^f^* Living near a high traffic road was missing for one household. *^g^* Cast iron was missing for one household. *^h^* Aluminum pots, pans, and dishes used at home was missing for 2 households.

**Table 4:** Factors associated with blood manganese concentrations based on univariable General Linear Models (*N* = 60).

Exposure Variables	Category		Univariable				
			Yes		No		*p*-Value
			Geometric Mean Mn (μg/L) *	*N*	Geometric Mean Mn (μg/L) *	*N*	
Sex of child	Male		11.90	54	10.72	6	0.44
Maternal age *^a^* (at child’s birth)	More than 35 years		12.75	7	10.83	50	0.19
Paternal age *^b^* (at child’s birth)	More than 35 years		11.24	17	10.45	42	0.38
Parental education levels *^c^* (at child’s birth)	At least one of the parents had education beyond high school		10.85	36	10.77	14	0.94
Socioeconomic status (SES)	High SES (own a car)		9.68	11	11.12	49	0.19
Source of drinking water *^d^*	Bottled water		10.61	51	11.44	3	0.66
Source of cooking water *^e^*	Piped water		10.37	47	10.57	5	0.88
Fruits and vegetables consumption	Leafy vegetables	Lettuce	10.46	21	11.05	39	0.52
		Spinach, kale	10.04	29	11.53	31	0.09
	Fruits	Blackberry	9.82	13	11.14	47	0.20
		Banana	11.13	52	9.10	8	0.09
		Watermelon	10.71	34	11.00	26	0.75
		Other melons (cantaloupe, honeydew)	10.82	27	10.85	33	0.99
Seafood consumption	Imported seafood	Ate shrimp	11.05	5	10.82	55	0.89
	Fresh seafood	Ate channel catfish	11.35	3	10.81	57	0.80
		Ate lake/pond fish (catfish, crappie)	10.49	6	10.88	54	0.79
		Ate bay fish (speckled Trout, redfish, flounder)	10.64	11	10.88	49	0.83
		Ate river fish (bass, trout)	10.87	22	10.82	38	0.96
		Ate offshore fish (tuna, snapper, whiting)	10.96	5	10.83	55	0.93
		Ate shellfish (lobster, crab, crawfish)	20.00	1	10.72	59	0.05
Grain/Starches consumption	Dark bread		10.48	21	11.03	39	0.55
	Oatmeal		11.86	10	10.64	50	0.32
	Cold breakfast (cereal)		10.77	41	10.98	19	0.82
	Pasta, macaroni, noodles		10.60	50	12.10	10	0.22
	Biscuits		10.87	59	8.90	1	0.53
	Polenta		11.48	37	9.88	23	0.07

* Mean Mn indicate the geometric mean = Exp. [Mean (lnMn)]; The **Yes** column includes children who met the category specified in front of each exposure variable or the food consumed. The **No** column includes children who did not meet the category specified in front of each exposure variable or never consumed the food. **^a^** Maternal age was missing for 3 mothers. ^b^ Paternal age was missing for 1 father. *^c^* Parental education levels were missing for 10 parents *^d^* Source of drinking water was missing for 6 households. *^e^* Source of cooking water was missing for 8 households.

**Table 5:** Factors associated with blood aluminum concentrations based on univariable General Linear Models (*N* = 60).

Exposure Variables	Category		Univariable				
			Yes		No		*p*-Value
			Geometric Mean Al (μg/L) *	*N*	Geometric Mean Al (μg/L) *	*N*	
Sex of child	Male		14.53	54	13.67	6	0.91
Maternal age *^a^* (at child’s birth)	More than 35 years		13.83	7	14.39	50	0.94
Paternal age *^b^* (at child’s birth)	More than 35 years		15.51	17	13.20	42	0.64
Parental education levels *^c^* (at child’s birth)	At least one of the parents had education beyond high school		15.98	36	15.17	14	0.89
Socioeconomic status (SES)	High SES (own a car)		20.73	11	13.31	49	0.28
Source of drinking water *^d^*	Bottled water		15.80	51	9.80	3	0.50
Source of cooking water *^e^*	Piped water		6.51	47	17.19	5	0.08
Types of toys the child plays with	Plastic		14.00	59	88.00	1	0.14
	Electronic		14.58	44	14.06	16	0.92
	Battery operated		15.41	51	9.99	9	0.33
	Stuffed		19.18	15	13.14	45	0.30
Types of pots, pans, and dishes used at home	Cast iron *^f^*		18.76	18	13.32	41	0.32
	Teflon		14.68	55	12.09	5	0.74
	Aluminum *^g^*		13.11	29	15.32	29	0.64
Fruits and vegetables consumption	Leafy vegetables	Lettuce	13.34	21	15.07	39	0.72
		Spinach, kale	18.31	29	11.89	31	0.18
	Fruits	Blackberry	13.60	13	14.68	47	0.84
		Banana	15.23	52	10.21	8	0.39
		Watermelon	17.57	34	11.17	26	0.16
		Other melons (cantaloupe, honeydew)	20.47	27	10.86	33	0.04
Seafood consumption	Imported seafood	Ate shrimp	44.95	5	13.02	55	0.03
	Fresh seafood	Ate channel catfish	32.08	3	13.85	57	0.25
		Ate lake/pond fish (catfish, crappie)	9.92	6	15.06	54	0.43
		Ate bay fish (speckled trout, redfish, flounder)	17.09	11	13.90	49	0.62
		Ate river fish (bass, trout)	11.25	22	16.69	38	0.23
		Ate offshore fish (tuna, snapper, whiting)	21.08	5	13.95	55	0.47
		Ate shellfish (lobster, crab, crawfish)	27.00	1	14.29	59	0.61
Meat consumption	Beef		**16.06**	**42**	**11.27**	**18**	**0.31**
	Lamb		32.06	9	12.54	51	0.03
	Goat		37.90	4	13.48	56	0.10
	Pork		14.06	44	15.55	16	0.78
	Minced meat (beef/pork/chicken)		14.15	47	15.52	13	0.81
Juices/ Soft Drink	Juices (e.g., orange, tomato, etc.)		12.22	45	23.84	15	0.07
	Flavored beverages		15.49	24	13.78	36	0.72
	Soft drinks (soda, Coke/Pepsi)		29.94	7	13.11	53	0.09
	Hot tea (e.g., black, Earl Grey, green)		13.95	27	14.86	33	0.84
	Iced tea		19.05	9	13.75	51	0.46

* Mean Al indicate the geometric mean = Exp. [Mean (lnAl)]. The **Yes** column includes children who met the category specified in front of each exposure variable or the food consumed. The **No** column includes children who did not meet the category specified in front of each exposure variable or never consumed the food. **^a^** Maternal age was missing for three mothers. ^b^ Paternal age was missing for one father. *^c^* Parental education levels were missing for 10 parents *^d^* Source of drinking water was missing for six households. *^e^* Source of cooking water was missing for eight households. *^f^* Cast iron was missing for one household. *^g^* Aluminum pots, pans, and dishes used at home was missing for two households.

## Abbreviations

Lead	Pb
Mercury	Hg
Cadmium	Cd
Arsenic	As
Manganese	Mn
Aluminum	Al
Autism Spectrum Disorder	ASD
Typically developing	TD
*glutathione S-transferase pi 1*	*GSTP1*
World Health Organization	WHO
Intelligence quotient	IQ
University of Medicine and Pharmacy of Carol Davila	UMF Carol Davila
The University of Texas Health Science Center at Houston	UTHealth
Diagnostic and Statistical Manual of Mental Disorders, Fourth Edition	DSM-IV
International Classification of Diseases, 10th Revision	ICD-10
Autism Disorder Observation Schedule	ADOS
Autism Diagnostic Interview—Revised	ADI-R
Socioeconomic status	SES
Michigan Department of Health and Human Services	MDHHS
Agency for Toxic Substances and Disease Registry	ATSDR
Centers for Disease Control and Prevention	CDC
Laboratory Corporation of America	LabCorp
Natural Logarithm	ln
Limits of Detection	LoD
General Linear Models	GLMs
Geometric mean	GM
Attention-deficit hyperactivity disorder	ADHD
Environment and Health Information System	ENHIS
